# Echocardiographic Assessment of Preload Responsiveness in Critically Ill Patients

**DOI:** 10.1155/2012/819696

**Published:** 2011-09-12

**Authors:** Alexander Levitov, Paul E. Marik

**Affiliations:** Division of Pulmonary and Critical Care Medicine, Eastern Virginia Medical School, Norfolk, VA 23507, USA

## Abstract

Fluid challenges are considered the cornerstone of resuscitation in critically ill patients. However, clinical studies have demonstrated that only about 50% of hemodynamically unstable patients are volume responsive. Furthermore, increasing evidence suggests that excess fluid resuscitation is associated with increased mortality. It therefore becomes vital to assess a patient's fluid responsiveness prior to embarking on fluid loading. Static pressure (CVP, PAOP) and echocardiographic (IVC diameter, LVEDA) parameters fails to predict volume responsiveness. However, a number of dynamic echocardiographic parameters which are based on changes in vena-caval dimensions or cardiac function induce by positive pressure ventilation or passive leg raising appear to be highly predictive of volume responsiveness.

## 1. Introduction

Shock (hemodynamic failure) is ubiquitous in the modern intensive care unit (ICU). Venodilation, transudation of fluid from the vascular space into the interstitium and increased insensible losses result in hypovolemia early in the course of patients with sepsis. Early goal-directed therapy (EGDT) emphasizes aggressive fluid resuscitation of septic patients during the initial 6 hours of presentation [1]. Persistent hypotension after initial fluid resuscitation is common and poses the dilemma of whether the patient should receive additional fluid boluses or a vasopressor agent should be initiated. Persistent signs of organ hypoperfusion such as oliguria make this decision crucial. While number of technologies including pulse counter analysis [2], trans-pulmonary thermodilution [3] and bioreactance [4] have all shown promise in evaluation of volume status of septic patients, bedside ultrasonography has already established itself as useful technique to evaluate cardiac function [5]. Applying the same echocardiographic techniques to dynamically assess the physiological response to spontaneous or mechanical ventilation, bedside maneuvers and the response to therapeutic interventions will likely become a cornerstone of hemodynamic monitoring in the modern ICU.

## 2. Benefits and Pitfalls of Fluid Resuscitation

 When hypovolemia (either absolute or relative) is suspected, fluid resuscitation will provide benefit to the patient by increasing venous return, cardiac output, arterial blood pressure and ultimately tissue perfusion. The rapidity, with which euvolemia is reestablished may be a decisive factor in the eventual outcome [1]. That being said, there is an increasing body of evidence suggesting that fluid resuscitation is not without serious and possibly lethal complications. Those complications may be related to preexisting conditions such as systolic or diastolic heart failure, cor pulmonale, or the development of sepsis-related cardiac dysfunction [6]. Extravasation of fluids may result in worsening of acute respiratory distress syndrome (ARDS) and prolonged mechanical ventilation [7]. Anemia and clotting disorders occur with hemodilution. Excessive fluid resuscitation can be positively correlated with increased mortality in the ICU [8–10]. Given the risk to benefit ratio of volume expansion, the key question is whether the patient would benefit from additional fluid boluses. It is essential to make this determination as clinical studies have repeatedly demonstrated that only about 50% of hemodynamically unstable ICU patients are volume responsive (see definitions below). 

## 3. Fluid Challenge versus Volume Responsiveness

Previously this question was answered by administering a “fluid challenge” of 30 mL/kg of crystalloid solution, and the patients clinical (blood pressure, heart rate, urine output) and hemodynamic response (CVP, PAOP) to the challenge was evaluated. Importantly, because a fluid challenge has to be given to assess volume responsiveness, and hypervolemia is associated with significant complications, one would suggest that the increase in mortality associated with invasive hemodynamic monitoring [11] may be attributed to this approach. Therefore, given the increased mortality associated with excessive fluid resuscitation it seems prudent to be able to predict the response to a fluid bolus prior to administering the bolus; a concept known as volume responsiveness. 

 The standard definition of volume responsiveness is a >15% increase in cardiac output in response to volume expansion. Although the volume of the fluid bolus has not been well standardized, a volume of between 500 mL to 1000 mL of crystalloid solution has been most studied. One or more baseline hemodynamic parameters are measured and evaluated for the ability to discriminate between responders and nonresponders.

## 4. Static Parameters

A static parameter is measured under a single ventricular loading condition and is presumed to reliably estimate the preload of the right ventricle (RV), left ventricle (LV), or both ventricles. This estimation is used to evaluate the probability of responsiveness to ventricular filling, by assuming that a lower preload increases the probability of a response to volume expansion. Several static parameters of ventricular preload have been used in the ICU; some are based on direct pressure measurements, while others use echocardiographic indices. 

### 4.1. Static Pressure Parameters

The traditional approach to fluid resuscitation consists of measuring a pressure parameter such as the central venous pressure (CVP) or pulmonary artery occlusion pressure (POAP) together with a cardiac output determination. The clinician would then prescribe a “fluid challenge” and reassess the above mentioned parameters. This approach has been largely discredited by the data suggesting a poor or no correlation between the CVP or PAOP and volume responsiveness as well as intravascular volume [12]. Nevertheless, the vast majority of intensivists still utilize the CVP to assess volume status [13] and the major critical care societies advocate for CVP as a measure of successful fluid resuscitation [14]. Several studies have demonstrated that the response to a fluid challenge even in healthy volunteers cannot be predicted by either the CVP or PAOP. In a study by Kumar et al. [15] in healthy subjects, static indices of ventricular preload (CVP,PAOP, LVEDV index, and RVEDV index) and cardiac performance indices (cardiac index, stroke volume index) were measured before and after 3 liters of normal saline loading. In this study, there was no correlation between baseline static pressure parameters and changes in the cardiac performance indices (cardiac index and stroke volume index) after fluid loading. Similarly, there was no correlation between changes in the CVP and PAOP and changes in cardiac performance [16]. A meta-analysis by Coudray et al. [17] reviewed five studies on a mixed population of spontaneously breathing critically ill patients and demonstrated the absence of a correlation between the initial PAOP and the response to a crystalloid infusion (an average of 1 liter).

### 4.2. Static Echocardiographic Parameters

As echocardiography is noninvasive, it has advantages over pressure-derived parameters particular those obtained from pulmonary artery catheterization. Transthoracic echocardiography (TTE) is preferred; however, in certain circumstance transesophageal echocardiography (TEE) may be required. The CVP and PAOP (left atrial pressure) can be approximated by echocardiography. In spontaneously breathing patients, there is a fairly good correlation between the size of the IVC and the CVP. However, Feissel et al. demonstrated that the absolute IVC size failed to predict fluid responsiveness in patients with septic shock [18]. 

**Figure 1:**
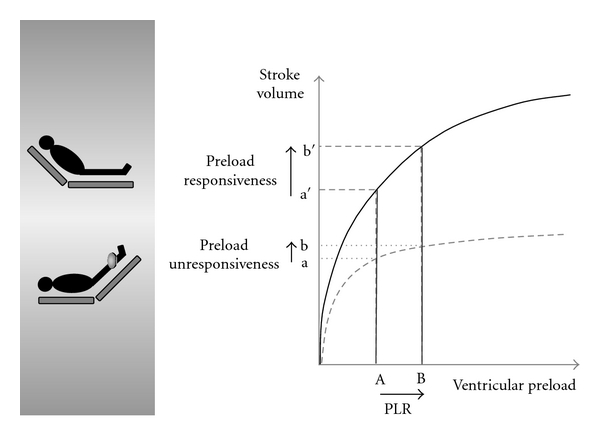
Depending on LV systolic function two distinct families of Frank-Starling relationships are formed, exemplified by solid and interrupted lines. Patients with hemodynamics following solid line pattern (preserved left ventricular systolic function) are more likely to benefit from preload manipulation, then those following the interrupted line pattern (reduced left ventricular systolic function). When Ventricle is functioning on the steep part of the Frank-Starling curve, there is a preload reserve. The passive leg raising (PLR) test (and a fluid challenge) increases stroke volume. By contrast, once the ventricle is operating near the flat part of the curve, there is no preload reserve and PLR (and a fluid challenge) has little effect on the stroke volume.

PAOP (left atrial pressure) estimates involve the use of Doppler mitral flow E/A ratio, pulmonary venous flow, tissue Doppler (E/Ea ratio), or colored coded Doppler (E/Vp ratio). While beyond the expertise level of most American intensivists, the estimated left atrial pressure can be estimated as part of a comprehensive echocardiographic examination performed by an experienced operator. However, it is worth noting that the PAOP fails to predict volume responsiveness whether measured directly or by echocardiography. The RV and LV diastolic diameter or area has been used as a measure of preload. However, Tavernier et al. and Feissel et al. [19, 20] have demonstrated that LV size (left ventricular end diastolic area LVEDA) is not a useful predictor of fluid responsiveness in patients on mechanical ventilation, unless LV is very small and hyperkinetic. A meta-analysis by Marik et al. [21] demonstrated the failure of the LVEDA to predict volume responsiveness in mechanically ventilated patients. Generally speaking, static parameters appear to be poor predictors of volume responsiveness except in patients with relatively obvious hypovolemia, which is a relatively uncommon event in modern ICU practice. It can, therefore, be concluded, that standard static indices of preload are not useful in predicting volume responsiveness in ICU patients. This observation may be due to dynamic changes in left (LV) and to a lesser degree right ventricular (RV) compliance, making the diastolic pressure-volume relationship nonlinear, unpredictable, and perhaps subject to change during resuscitation itself. Systolic left ventricular function is also a subject to change in critically ill patients, even those, without preexistent cardiac disease. Vieillard-Baron and coauthors demonstrated the development of systolic left ventricular dysfunction in 60% of patient with septic shock [6]. Changing left ventricular function makes it difficult to predict the position of the patient on his/her Frank-Starling curve. It is even difficult to estimate which family of Frank-Starling relationships should be utilized to predict fluid responsiveness (see Figure 1). Furthermore, the development of acute right ventricular failure (acute cor pulmonale), particularly in patients receiving mechanical ventilation with high plateau pressures (>27 cm H_2_O), further confounds the issue [22]. Unrecognized acute right ventricular failure can mimic hypovolemia hemodynamically but would not respond or even get worse with volume expansion. Dynamic hemodynamic parameters offer the intensivists the best opportunity of predicting response to fluid resuscitation.

## 5. Dynamic Parameters of Volume Responsiveness

Dynamic parameters are used to determine the patients position on his/her Frank-Starling curve (Figure 1) and specifically to determine whether the patient is situated on the ascending portion of the Frank-Starling curve where an increase of preload results in increase of stroke volume (SV) (preload-dependent situation), or on the plateau portion where a variation of preload does not alter SV (preload-independent situation). Several approaches can be used to determine on what portion of the preload/stroke volume relationship the ventricle is functioning to establish the diagnosis of preload dependence or independence. Most utilize observation of cardiac response to either mechanical or spontaneous breathing cycle and breathing related variations in intrathoracic pressure. These pressure changes directly effect RV and LV preload and provides a tool to correlate these preload changes to SV. Alternatively, bedside maneuvers such as passive leg raising (PLR) result in alterations of RV and LV preload can be utilized to establish similar correlations.

### 5.1. Ventilated versus Spontaneously Breathing Patient

By significantly increasing RV preload, spontaneous breathing is crucial to maintaining normal hemodynamic status. Mechanical ventilation substantially increases intrathoracic pressure, decreasing RV preload and thus has predictably negative hemodynamic consequences. Moreover, traditional positive pressure ventilation also reverses inspiration/expiration phases from a hemodynamic point of view, changing many breathing related phenomena (i.e., paradoxical pulse) to its opposite (reverse pulsus paradoxus) [23]. 

### 5.2. Dynamic Echocardiographic Parameters in Patients on Mechanical Ventilation

**Figure 2:**
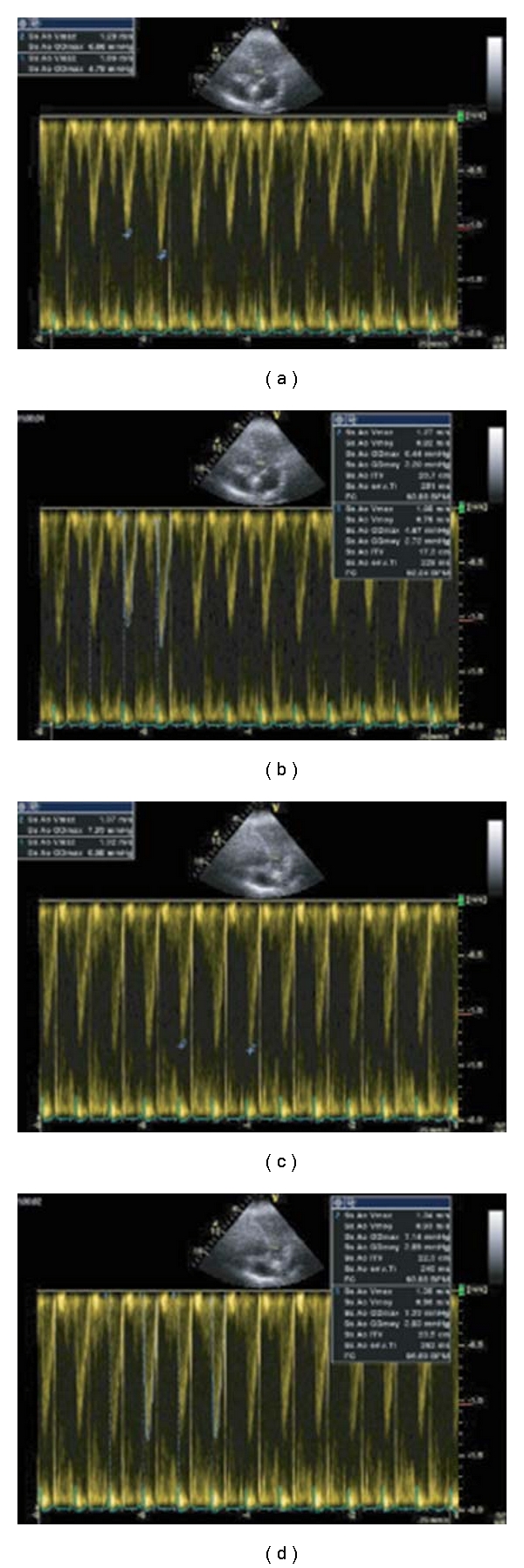
Respiratory variations of maximal velocity (Vmax) (a and c) and VTI (b and d) of aortic blood flow recorded with a pulsed Doppler transthoracic echocardiography in a mechanically ventilated patient (a and c). Presence of significant respiratory variations of Vmax (Vmax − Vmin/[Vmax + Vmin/2]; 1.29 − 1.09/1.19 = 17%) and VTI (VTImax − VTImin/[VTImax + VTI min/2]; 20.7 − 17.3/19 = 18%). (b and d) Same patient after volume expansion, regression of the respiratory variations: Vmax (1.37 − 1.32/1.34 = 4%), VTI (23.5 − 22.3/22.9 = 5%). Reproduced with permission from Levitov et al. “Critical care Ultrasonography” Mc Graw Hill 2009.

**Figure 3:**
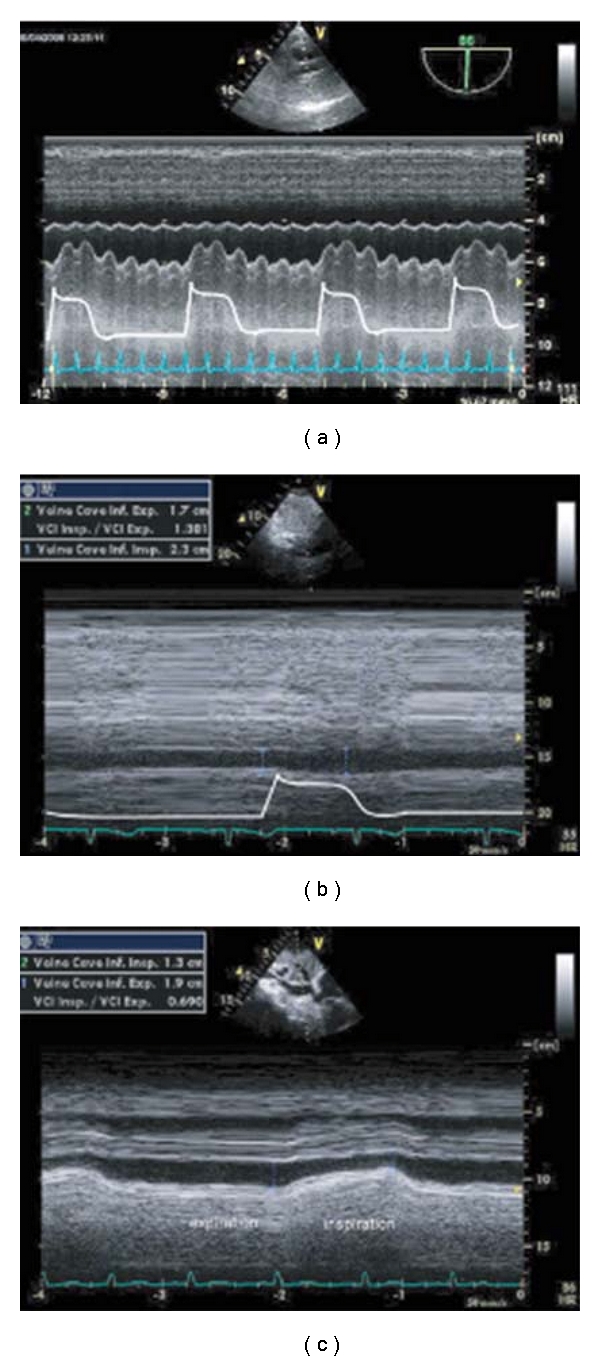
Respiratory vena cava variations in different circumstances. (a) Significant superior vena cava (SVC) collapsibility recorded with transesophageal echocardiography (TEE). (b) Significant inferior vena cava (IVC) distensibility recorded with transthoracic echocardiography (TTE) in a mechanically ventilated patient. (c) Significant vena cava collapsibility recorded with transthoracic echocardiography (TTE) in a spontaneously breathing patient. Reproduced with permission from Levitov et al. “Critical care Ultrasonography” Mc Graw Hill 2009.

Analysis of the respiratory changes of LV stroke volume during mechanical ventilation provides a dynamic, biventricular evaluation of preload dependence. The respiratory changes of stroke volume can be estimated by Doppler analysis of velocity-time integral (VTI) during TTE or TEE. In clinical studies, maximal ascending aortic flow velocity or VTI variation measured with TEE predict, with high sensitivity and specificity, increases in cardiac output after fluid infusion in patients with septic shock. A cut-off value of respiratory cycle changes of 12% for maximal flow velocity and 20% for aortic VTI-discriminated responders from nonresponders [20]. Similar information that can be obtained from interrogation of ascending aorta with TTE (Figure 2) or descending aorta. Another approach to identify volume responsiveness used 2D images. Cannesson et al. [24] assessed LV diastolic area (LVDA) changes by TEE from the short axis view. They found that a 16% respiratory variation of LVDA predicted fluid responsiveness with a sensitivity of 92% and a specificity of 83%. Utilizing a similar principle, IVC and superior vena cava (SVC) diameter changes during mechanical ventilation can be used to predict fluid responsiveness (see Figure 3). The inferior vena cava diameter by TTE is analyzed from a subcostal long axis view and recorded by using M mode. The superior vena cava diameter is recorded from TEE longitudinal view at 90–100°. Cut-off values of 12% (by using (max − min)/mean value)) and 18% (by using (max − min)/min value) for IVC (distensibility index) and 36% for SVC (collapsibility index) were found to accurately (sensitivity 90%, specificity 100%) separate responders and non-responders that as an intrathoracic. The potential benefit of using SVC is due to the fact that as intrathoracic organ the SVC is subject to greater respiratory variations and intrathoracic pressure resulting from mechanical ventilation. Though SVC collapsibility appears to be the most “reliable index of volume responsiveness”, it does require TEE [25] and thus is out of reach of most intensivists in the United States. 

Ventilator induced preload changes as predictors of volume responsiveness have only been evaluated in patients on flow limited, volume cycled ventilation and without patient ventilator dyssynchrony. Furthermore, although the level of positive end expiratory pressure (PEEP) is known to influence venous return and biventricular function the effect of PEEP on echocardiographic assessment of volume responsiveness has not been studied. Other requirements include presence of a normal sinus rhythm, normal intra-abdominal pressure and absence of significant RV dysfunction. Although a positive response to PLR seems to be predictive of volume responsiveness in mechanically ventilated patients (sensitivity 90% specificity 83%) [26] further studies are necessary to better understand the role of this bedside maneuver in this population of critically ill patients.

### 5.3. Dynamic Echocardiographic Parameters in Spontaneously Breathing Patients

**Figure 4:**
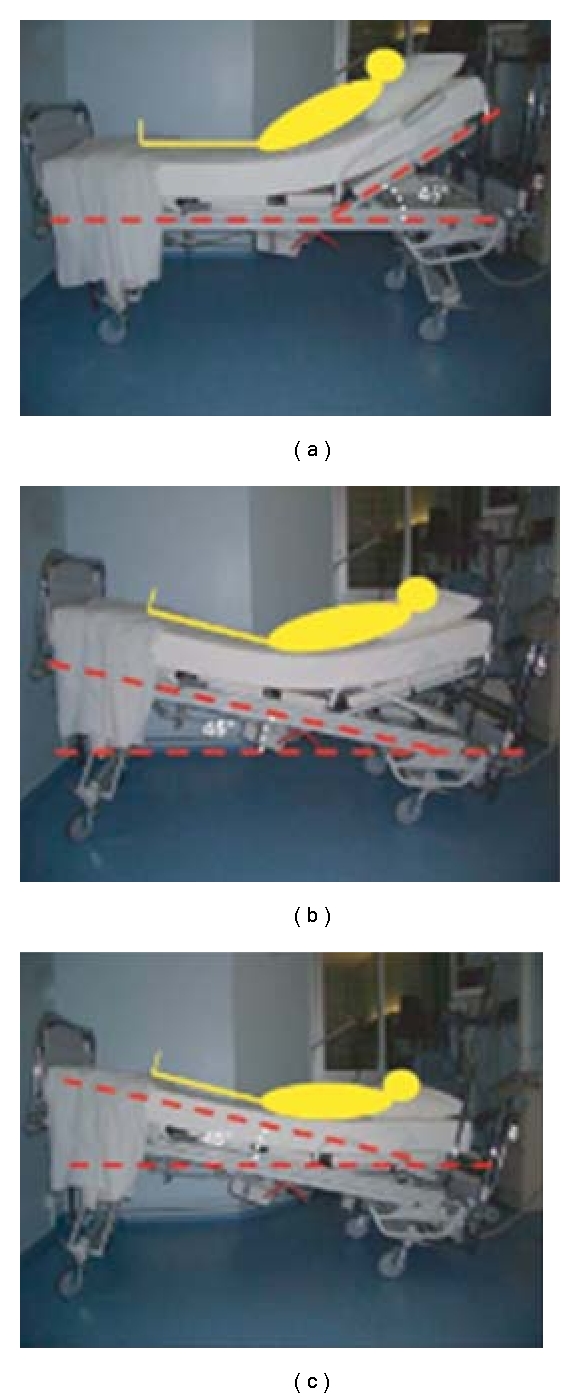
The realization of a passive leg raising maneuver in three steps: (a) at baseline the patient is laying in a semirecumbent position, the trunk of the patient at 45° up to the horizontal; (b) the entire bed is pivoted to obtain a head down tilt at 45°. (c) The head of the bed is adjusted to obtain a strictly horizontal.

Several publications have proposed using PLR maneuver to predict preload responsiveness (Figure 4). This maneuver rapidly mobilizes about 300–500 mL of blood from the lower limbs to the intrathoracic compartment and reproduces the effects of similar volume fluid bolus (Figure 1). Being completely reversible this maneuver is devoid of any risks associated with an actual “fluid challenge.” The test consists of raising both legs of the supine patient to an angle of 45° in relation to the bed while measuring SV and cardiac output before and immediately (1–3 minutes) following the PLR maneuver. This may be accomplished by measuring the VTI of the aortic outflow with either TTE (apical five-chamber view) or TEE (deep-gastric view). Monnet et al. [27] demonstrated that when PLR induced an increase of aortic flow of *>*10%, it was predictive of an increase of aortic flow of *>*15% in response to volume expansion (sensitivity: 97%; specificity: 94%). Volume expansion was performed with 500 mL of isotonic saline over 10 minutes. Thirty-seven (52%) of the 71 patients included in this study responded to volume expansion; 22 subjects had spontaneous breathing activity (spontaneous breathing mode with inspiratory assistance). This study also evaluated respiratory cycle induced pulse pressure variations. The authors concluded that respiratory cyclic variations of pulse pressure ≥12% were similarly predictive of an increase of aortic flow by *>*15% in response to volume expansion in mechanically ventilated patients (sensitivity: 88%; specificity: 93%). However, in spontaneously breathing patient's predictive value of respiratory pulse pressure variations was poor. In two other studies aortic VTI, stroke volume and cardiac output were recorded using transthoracic echocardiography in spontaneously breathing patients during a PLR maneuver. Lamia et al. [28] demonstrated a PLR-induced increase in stroke volume of 12.5% or more predicted an increase in stroke volume of 15% or more after volume expansion, with a sensitivity of 77% and a specificity of 100%. In this study, patients were intubated with spontaneous breathing. Static indices of preload such as left ventricular diastolic area and E/Ea ratio failed to predict volume responsiveness. Maizel et al. [29] studied 34 spontaneously breathing patients; an increase of cardiac output or stroke volume by *>*12% during PLR was highly predictive of volume responsiveness. Sensitivity and specificity values were 63% and 89%, respectively. In addition, this study demonstrated that PLR may be used to predict volume responsiveness in patients with atrial fibrillation. Increased intraabdominal pressure, however, strongly interferes with the ability of PLR to predict fluid responsiveness [30].

In conclusion, echocardiography provides the intensivist with several methods to determine volume responsiveness in patients with hemodynamic failure. The clinician with basic skills in critical care echocardiography may use respiratory variation of IVC diameter to identify the preload-dependent patient combined with pattern recognition of small hyperdynamic LV. The intensivist with advanced TTE skill level may use respiratory variation of SV determined by Doppler echocardiography (VTI) and changes in SV following the PLR maneuver to identify volume responsiveness. Intensivist with TEE skills may effectively utilize this modality in patients presenting technical challenge for TTE. Advent of minimally invasive TEE monitoring probes might allow intensivist views of SVC not available on TTE and real time LV and RV function monitoring abilities, previously unavailable at bedside. Widespread use of newer modes of mechanical ventilation (APRV, HFOV) provides new challenges and opportunities for the evaluation of their effect of cardiac performance and volume responsiveness. Further studies are necessary to determine if this increase in physiological insight will translate into improved outcomes of critically ill patients.

##  Conflict of Interests

The authors declare that they have no conflict of interests.
